# Sectional Impression Tray With Reusable Handle: A Single Solution for All Microstomia Cases

**DOI:** 10.7759/cureus.36038

**Published:** 2023-03-12

**Authors:** Shreya Colvenkar, Himaja Swayampakula, Jayasri Vanapalli, Sri Varsha Tirukovalur, Ramesh Kunusoth

**Affiliations:** 1 Department of Prosthodontics, MNR Dental College and Hospital, Sangareddy, IND; 2 Department of Oral and Maxillofacial Surgery, MNR Dental College and Hospital, Sangareddy, IND; 3 Department of Oral Medicine and Radiology, MNR Dental College and Hospital, Sangareddy, IND

**Keywords:** impression, reusable, magnets, microstomia, impression tray, sectional

## Abstract

A dimensionally accurate impression is one of the primary determinants for the precise fabrication of complete denture prostheses in microstomia patients. This can be achieved with the help of sectional trays. This technical report describes the fabrication of a sectional impression tray with a reusable sectional handle using magnets. The handles can be sterilized and reused, thus saving clinical time for future use. The proposed method provides ease of reassembling and disassembling, easy placement, and guided orientation of the two tray segments.

## Introduction

Microstomia is a condition that affects the size of the oral orifice, resulting in an abnormally small opening. It can be caused by a variety of conditions such as surgery, trauma, burns, radiotherapy, or temporomandibular joint diseases [1-3]. While it is not a disease in itself, it can lead to other medical issues, including difficulty with eating and speaking. Microstomia can significantly impact the quality of life and should be treated as soon as possible to minimize any long-term effects.

The insertion of a standard stock and custom impression tray presents initial difficulty in prosthetic rehabilitation. Various sectional impression trays are described in the literature [4-9]. These sectional tray designs are successful in making an accurate impression, but every time a new case comes to the hospital, a new tray design needs to be planned instead of using a standardized design for all patients.

The purpose of this article is to describe the standardized technique for the fabrication of sectional dentulous stock impression trays and edentulous custom impression trays for multiple microstomia patients using the reusable metallic sectional handle. Fabricated sectional handles can be sterilized and reused, thus saving clinical time for future use.

## Technical report

Fabrication of a tray handle

**Figure 1:**
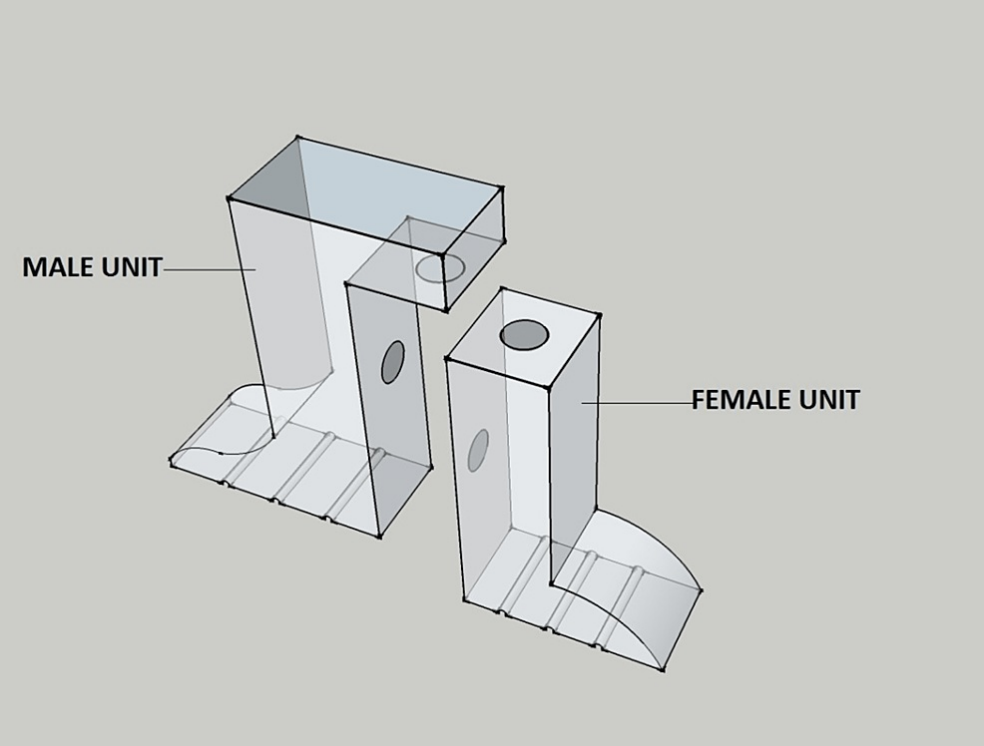
Sectional handle

The handle has two parts, the male and the female unit (Figure 1).

Male unit: It has a vertical flange measuring 4x4x3mm with a horizontal plate measuring 8x3x4mm. The undersurface of the horizontal plate and the inner surface of the vertical flange has a 3-mm circular depression.

Female unit: It has a vertical flange measuring 5x4x3mm. The vertical flange carries a 3-mm circular depression on the superior and inner surfaces.

Sectional units have a horizontal plate with grooves on the undersurface to attach the acrylic resin.

**Figure 2:**
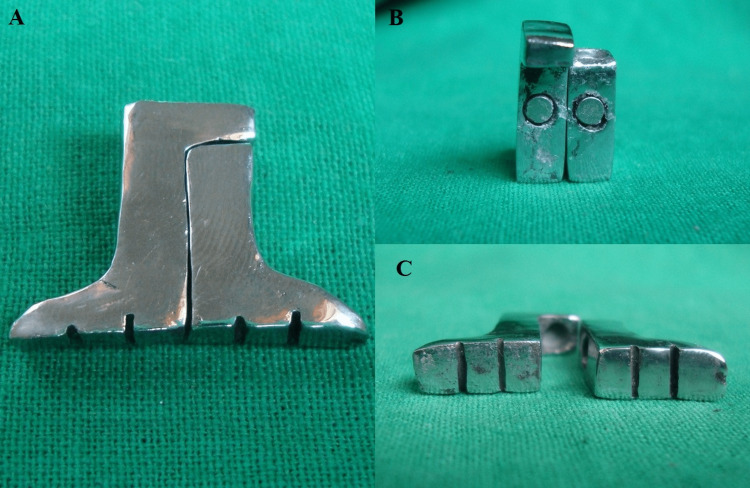
A: interlocked metal sectional handle; B: sectional handle showing magnet; C: inner view showing grooves

The above-mentioned design was fabricated in an inlay wax (Pyrax inlay wax; Pyrax Eports, Uttarakhand, India) and cast in a base metal alloy (Wirolloy® NB, Bego, Germany) (Figure 2).

After finishing and polishing, attach stainless steel-encased iron neodymium boron magnets (Rare Earth Magnet, Permag Products Pvt. Ltd., Pune, India), measuring 3mm in the depression with cyanoacrylate adhesive.

Modification of dentulous stock impression tray

**Figure 3:**
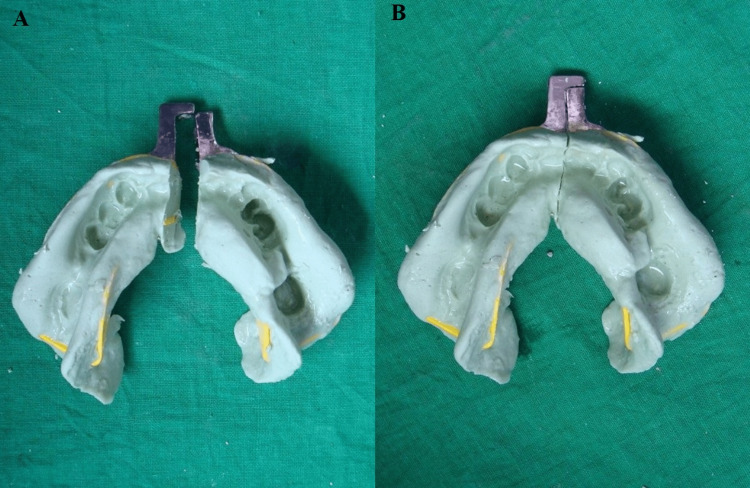
Impression with irreversible hydrocolloid A: sectional tray; B: interlocked tray

Modify the preselected perforated plastic stock impression tray by removing the handles and sectioning it along the midline. The female unit of the handle is attached to the anterior region of the first half of the tray with the auto-polymerized acrylic resin. Once the material sets, the male unit of the handle is attached to the remaining half of the tray with an auto-polymerized acrylic resin such that both units are properly aligned. Load one half of the stock tray with irreversible hydrocolloid impression material and make the impression. Remove the tray once the impression material is set. Trim the excess impression material such that it is well flushed against the edge of the tray. Load the other half of the stock impression tray with irreversible hydrocolloid and make the impression. Once the impression material sets, remove each half separately intraorally and join it together externally (Figure 3).

Fabrication of custom impression tray

**Figure 4:**
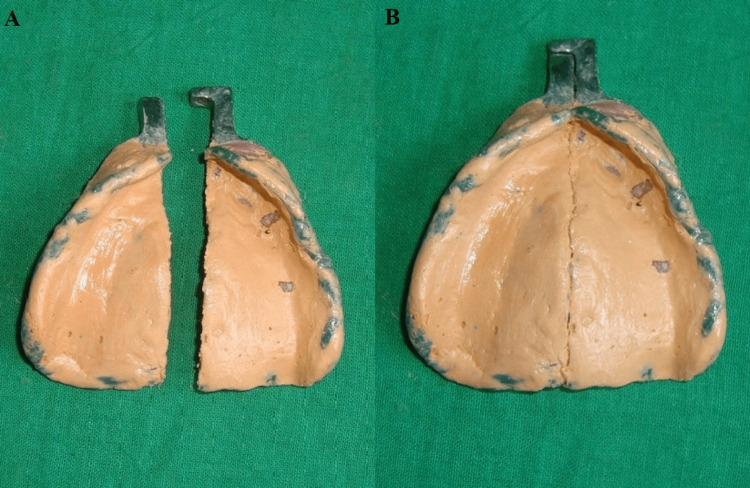
Secondary impression A: sectional custom tray; B: interlocked custom tray

Fabricate the complete maxillary and mandibular custom impression tray using auto-polymerized acrylic resin, and then section the tray along the midline. Attach the female unit of the handle with an auto-polymerized acrylic resin in the anterior aspect of one half of the tray. Once set, attach the male unit with auto-polymerized acrylic resin to the other half of the custom impression tray such that the two units are properly aligned. Carry out sectional border molding for the right and left halves of the tray with low-fusing impression compound (Pinnacle Tracing Sticks; Dental Products of India Ltd, Mumbai, India). Place relief holes in the right and left half of the tray after the removal of the wax spacer. Apply separating media along the edge of the right and left half of the tray along the midline. Add zinc oxide eugenol impression paste on the right half of the tray and make an impression. Remove the tray once the impression material is set. Trim excess impression material such that it is well flushed against the edge of the tray. Add zinc oxide eugenol impression paste on the left half of the tray and make an impression. Separate the sectional trays intraorally once the impression material sets and join it externally (Figure 4).

## Discussion

A dimensionally accurate impression is one of the primary determinants for the precise fabrication of complete denture prostheses in microstomia patients. This can be achieved with the help of sectional trays. A sectional tray with a reusable handle helps ease the placement and guided orientation of the two tray segments. The added advantage is that it’s simple to use and provides the ease of separating and rejoining the tray in the patient's mouth. Hence, a rigid nonelastic impression material was used. Both elastomeric and non-elastomeric impression materials were used in a similar technique [8-9].

Sectional handles were fabricated using magnets since their strong attractive force forms a stable assembly in all directions once the right and left units are interlocked. If magnets are not available, different interlocking attachment designs can be fabricated in the handle itself, but care needs to be taken that it has to be done with a lot of precision to prevent misalignment of the two sections.

Lego blocks [4,7] and locking levers [9] were used for the fabrication of mandibular sectional impression trays, but their use in maxillary sectional impression trays was questionable. It can also be used for both edentulous as well as dentulous patients for making an impression of both arches.

The disadvantage of this technique is that modification of metal stock trays is not as easy as compared to plastic stock trays.

## Conclusions

This clinical report describes the fabrication of a sectional tray with a reusable handle retained by magnets for a microstomia patient. A sectional impression tray provides ease of reassembling and disassembling and is stable in all directions, thus overcoming difficulties associated with making impressions in microstomia patients.
